# Endometriosis Is Associated with Functional Polymorphism in the Promoter of Heme Oxygenase 1 (*HMOX1*) Gene

**DOI:** 10.3390/cells10030695

**Published:** 2021-03-21

**Authors:** Łukasz Milewski, Aneta Ścieżyńska, Joanna Ponińska, Marta Soszyńska, Ewa Barcz, Piotr I. Roszkowski, Paweł Kamiński, Paweł Włodarski, Rafał Płoski, Jacek Malejczyk

**Affiliations:** 1 Department of Histology and Embryology, Centre for Biostructure Research, Medical University of Warsaw, 02-004 Warsaw, Poland; lmilewski@o2.pl (Ł.M.); asciezynska@wum.edu.pl (A.Ś.); marta.soszynska@onet.pl (M.S.); 2 Center for Preclinical Research and Technology, Medical University of Warsaw, 02-097 Warsaw, Poland; 3 Laboratory for Experimental Immunology, Military Institute of Hygiene and Epidemiology, 01-163 Warsaw, Poland; 4 Department of Medical Biology, National Institute of Cardiology, 04-628 Warsaw, Poland; j.poninska@icard.pl; 5 1st Department of Obstetrics and Gynecology, Medical University of Warsaw, 02-015 Warsaw, Poland; ewa.barcz@interia.pl; 6 2nd Department of Obstetrics and Gynecology, Medical University of Warsaw, 00-315 Warsaw, Poland; roszkowskip@wp.pl; 7 Department of Gynecology and Gynecological Oncology, Military Institute of Medicine, 04-349 Warsaw, Poland; pawel.kaminscy@gmail.com; 8 Department of Methodology, Medical University of Warsaw, 02-097 Warsaw, Poland; pawel.wlodarski@wum.edu.pl; 9 Department of Medical Genetics, Centre for Biostructure Research, Medical University of Warsaw, 02-106 Warsaw, Poland; rploski@wp.pl

**Keywords:** endometriosis, heme oxygenase 1, inflammatory response, single-nucleotide polymorphism

## Abstract

Endometriosis is a common gynecological disorder characterized by the ectopic growth of endometrial-like tissue outside the uterine cavity. Etiopathogenesis of endometriosis is poorly understood; it is plausible, however, that the disease may be associated with oxidative stress related to local heme and iron metabolism. Therefore, the aim of the study was to reveal a possible association of endometriosis with a stress-inducible heme oxygenase 1 (HMOX1). For this purpose, 228 patients with clinically confirmed endometriosis and 415 control parous women from general Polish population were examined for functional –413A>T (rs2071746) single-nucleotide polymorphism (SNP) and (*GT*)_n_ dinucleotide repeat length polymorphism in the promoter of *HMOX1* gene. In addition, –413A>T SNP was assessed by the specific TaqMan^®^ SNP Genotyping Assay, and (*GT*)_n_ polymorphism was determined by PCR product size analysis. We found that endometriosis is associated with an increased frequency of −*413A*(*GT*)_31,32_ haplotype (OR (95%CI) = 1.27 (1.01–1.60), *p* = 0.0381) and −*413A*(*GT*)_31,32_ homozygous genotype [OR (95%CI) = 1.51 (1.06–2.17), *p* = 0.0238]. These data suggest that endometriosis is associated with functional polymorphism of *HMOX1* gene, and this gene may play a part in the pathogenesis of this disorder.

## 1. Introduction

Endometriosis is a common gynecological disorder affecting from 2% up to 22% (approximately 10%) women in reproductive age [1]. It is associated with ectopic implantation and growth of endometrial-like tissue (endometrial glands and stroma) in the pelvic cavity. The disease manifests with pelvic inflammatory reactions and pain and is one of the major causes of female infertility [2,3]. Etiopathogenesis of endometriosis is still poorly understood. It appears to be a multifactorial trait with the involvement of a variety of genetic and environmental factors [3,4,5].

There exist several theories of development of endometriosis [2,5,6]. However, the most accepted one postulates that ectopic endometriotic tissue originates from retrograde menstruation [6,7]. However, it is still unclear what facilitates survival and heterotopic implantation of endometrial epithelial and stromal cells. This may be related to abrogated elimination of endometriotic cells by NK cells, their increased resistance to apoptosis, and increased expression of adhesion molecules, as well as facilitation of local angiogenic response [8,9,10,11,12]. The possible mechanisms of these phenomena, however, still remain to be elucidated.

There is a growing body of evidence that the mechanisms involved in etiopathogenesis of endometriosis may include the pathways responsible for regulation of local oxidative stress and detoxification [13,14,15]. In particular, the pathways involved in heme and iron metabolism may be of interest, especially since these agents were found to be increased in the peritoneal fluid of women with endometriosis and may be involved in the pathogenesis of this disorder [16,17,18,19,20,21]. Heme and iron metabolism depend on activity of heme oxygenases (HMOXs), rate-limiting enzymes responsible for heme degradation leading to generation of equimolar amounts of biliverdin, carbon monoxide (CO), and free iron [22,23,24]. A key enzyme is stress-inducible heme oxygenase 1 (HMOX1), a 32.8-kDa enzyme encoded in humans by *HMOX1* gene located on chromosome 22q12 [24,25,26].

Factors involved in the induction of HMOX1 include various oxidative agents and inflammatory cytokines, which strongly suggests that activity of this enzyme represents an adaptive response mechanism to cellular oxidative stress [26,27]. Expression of HMOX1 has been reported to depend on the functional polymorphisms in the 5′ UTR region promoter of *HMOX1* gene, namely –413A>T (rs2071746) single-nucleotide polymorphism (SNP) [28,29] and (*GT*)_n_ dinucleotide repeat length polymorphism [30]. This strongly implies that regulation and biological significance of *HMOX1* expression at least partially depends on the host genetic background.

HMOX1 has been found to be expressed in endometriotic lesions [17]. However, a relationship between this enzyme and development of endometriosis has not been established, so far. Therefore, the aim of the present study was to assess whether endometriosis is associated with known functional polymorphisms of *HMOX1* gene.

## 2. Materials and Methods

### 2.1. Patients and Control Subjects

All women with endometriosis and control subjects included in the present study were diagnosed at I and II Department of Obstetrics and Gynecology, Medical University of Warsaw and the Department of Gynecology, Military Institute of Medicine, Warsaw, Poland. The disease has been confirmed and classified on the basis of laparoscopic and histopathological examinations according to the revised criteria of the American Fertility Society (rAFS) [31].

The genetic association study included 228 Polish Caucasian women with endometriosis (mean age 33.1 ± 7.5, range 22–58 years). This group consisted of 11 (4.8%) patients with minimal/mild (rAFS stage I/II) and 217 (95.2%) patients with moderate/severe (rAFS stage III/IV) disease. Ovarian endometriotic cysts were present in 217 (95.2%) cases, and peritoneal lesions were found in 110 (48.2%) women. Both type of lesions was detected in 189 (82.9%) patients, 28 (12.3%) patients had only ovarian cysts, and 11 (4.8%) patients had only peritoneal lesions.

The control group for the genetic study consisted of 415 anonymous unrelated parous women randomly selected from the cohort representative of background population of Central Poland which has been described elsewhere [32].

The study has been approved and was conducted according to strict guidelines of the ethical committee at the Medical University of Warsaw and all patients gave an informed consent to the study.

### 2.2. Typing for −413A>T and (GT)_n_ Polymorphisms

Genomic DNA was isolated from 5 mL of the peripheral blood by the salting-out method [33]. The quality of isolated DNA was checked spectrophotometrically and the optical 260/280 nm density ratio at of the samples was always >1.7. DNA samples were tested for the −413A>T SNP (rs2071746) of *HMOX1* gene using specific TaqMan^®^ SNP Genotyping Assay and 7500 real-time PCR system with sequence-detection software (Applied Biosystems, Foster City, CA, USA).

The (*GT*)_n_ dinucleotide length polymorphism was determined by amplifying the 5′ flanking region of *HMOX1* gene containing the (*GT*)_n_ repeats using the FAM-labeled sense 5′-AGAGCCTGCAGCTTCTCAGA-3′ and an antisense 5′-ACAAAGTCTGGCCATAGGAC-3′ primers, as described by Kimpara et al. [34]. The amplicon size was determined by the 3130 Genetic Analyzer (Applied Biosystems) and Gene Mapper software (Applied Biosystems).

### 2.3. Statistical Analysis

Continues variables were analyzed by Mann–Whitney *U* test using Statistica 10.0 software (StatSoft Inc., Tulsa, OK, USA). Allele, haplotype, and genotype distributions were analyzed by Pearson’s chi-squared (χ^2^) test or, as appropriate, two-tail Fisher exact probability test using VassarStats statistical calculator available at http://vassarstats.net, accessed on 21 March 2021. Hardy-Weinberg equilibrium (HWE), linkage disequilibrium, and haplotype analysis was performed using PLINK [35] or Arlequin [36] software. Odds ratios (OR) and their 95% confidence intervals (95% CI) for the indicated genotype were calculated versus a pool of all other genotypes. The differences between the tested variables were considered significant at least at *p* < 0.05.

## 3. Results

The distribution of *HMOX1* –413 SNP *A* and *T* alleles and genotypes in patients with endometriosis and control population is shown in Table 1. The genotype distribution in control subjects was in Hardy–Weinberg equilibrium (*p* = 0.987). On the contrary, genotype distribution in women with endometriosis did not fulfill criteria for Hardy–Weinberg equilibrium (*p =* 0.004). An analysis of allele distribution did not reveal any differences between women with endometriosis and control group [OR (95%CI) = 1.16 (0.92–1.47), *p =* 0.20]. However, the frequency of –*413AA* genotype was significantly higher in endometriosis group (OR (95%CI) = 1.48 (1.06–2.07), *p =* 0.0228). Endometriosis was also associated with a significantly decreased frequency of –*413AT* genotype (OR (95%CI) = 0.66 (0.48–0.92), *p =* 0.0132) (Table 1).

**Figure 1:**
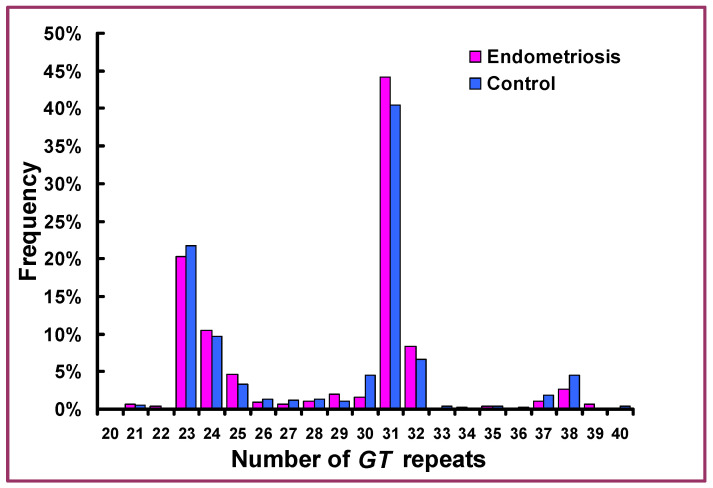
Frequency distribution of the length of *GT* repeats in the promoter of *HMOX1* gene in patients with endometriosis and control women.

Distribution of the number of *GT* repeats in the promoter of *HMOX1* gene in patients with endometriosis and control women is shown in Figure 1. As can be seen, the number of repeats ranged from 21 to 40, and the frequency distribution of (*GT*)_n_ alleles was uneven with many rare variants. When alleles with frequencies >5% were considered, two clearly different groups displaying 23 and 24 repeats as well as 31 and 32 repeats, respectively, could be distinguished. These two variant groups accounted for ca. 80% of all endometriosis and control cases, and therefore, only these groups were considered for specific analyses. They will be further referred to as (*GT*)_23,24_ and (*GT*)_31,32_ variants. The detailed results of distribution of (*GT*)_n_ alleles and genotypes in relation to −413A>T SNP genotypes are given in Table 2.

(*GT*)_n_ genotype distribution was in Hardy–Weinberg equilibrium both among endometriosis patients (*p =* 0.09) and control subjects (*p =* 0.39). Distribution of (*GT*)_23,24_ and (*GT*)_31,32_ variants and their genotypes are shown in Table 1. As can be seen, the frequency of (*GT*)_23,24_ in endometriosis and control groups was 30.8% and 31.6%, respectively, whereas the respective frequency of (*GT*)_31,32_ was 52.4% and 47.2%. The difference in distribution of (*GT*)_23,24_ in endometriosis and control group was not significant (OR (95%CI) = 0.96 (0.75–1.23), *p =* 0.777), whereas the difference in the frequency of (*GT*)_31,32_ had a borderline significance (OR (95%CI) = 1.23 (0.98–1.55), *p =* 0.0754). Analysis of distribution of (*GT*)_23,24_ and (*GT*)_31,32_ genotypes has revealed that endometriosis is associated with increased frequency of (*GT*)_31,32_ homozygotes (OR (95%CI) = 1.43 (1.01–2.05), *p =* 0.0480). There were no differences in distribution of (*GT*)_23,24_/(*GT*)_31,32_ heterozygotes and (*GT*)_23,24_ homozygotes in endometriosis group as compared with control (Table 1).

Haplotype analysis has revealed that *HMOX1* –413A>T SNP alleles and (*GT*)_n_ repeats are in linkage disequilibrium. In addition, –*413A* allele is associated with (*GT*)_31,32_ allele (r^2^ = 0.71), whereas –*413T* allele is associated with (*GT*)_23,24_ (r^2^ = 0.57).

Analysis of distribution of these two major *HMOX1* haplotypes and their related genotypes is presented in Table 3. As shown, endometriosis was significantly associated with –*413A*(*GT*)_31,32_ haplotype (OR (95%CI) = 1.27 (1.01–1.60), *p =* 0.0381). There was no difference in the frequency of –*413T*(*GT*)_23,24_ haplotype between endometriosis and control group (OR (95%) = 0.95 (0.67–1.35), *p =* 0.6954). Frequency of the other haplotypes was relatively low, and none of them showed any significant association with endometriosis (data not shown).

Analysis of distribution of –*413A* alleles in combination with (*GT*)_31,32_ variant is shown in Table 3. As demonstrated, –*413A*(*GT*)_31,32_/–*413A*(*GT*)_31,32_ homozygous genotype was significantly more frequent in women with endometriosis (OR (95%CI) = 1.51 (1.06–2.17), *p =* 0.0238). On the other hand, analysis of other combinations between –*413AA* homozygotes and other (*GT*)_n_ variants, as well as (*GT*)_31,32_(*GT*)_31,32_ homozygotes and –*413AT* or –*413TT* did not reveal any difference between endometriosis and control group (Table 3). None of the other *HMOX1* genotypes including combinations of –*413T* and (*GT*)_23,24_ alleles were significantly associated with endometriosis (data not shown).

## 4. Discussion

The results of our study show that endometriosis is related to functional polymorphism in the promoter of *HMOX1* gene. The disease was found to be associated with an increased frequency of both –*413AA* and (*GT*)_31,32_(*GT*)_31,32_ homozygotes. In addition, we observed that the distribution of –*413 HMOX* genotypes among patients (but not controls) significantly departed from Hardy–Weinberg equilibrium (HWE). Since departure from HWE among cases has been proposed as an independent test detecting an association [37,38], this observation further confirms that the *HMOX* locus is not neutral in endometriosis.

It should be mentioned, however, that both alleles are in linkage disequilibrium, and, indeed, endometriosis was associated with increased frequency of –*413A*(*GT*)_31,32_ haplotypes as well as –*413A*(*GT*)_31,32_/–*413A*(*GT*)_31,32_ genotypes. This indicates that disease may be related to homozygosity in either –*413A* or (*GT*)_31,32_ allele or may be linked to their combination. However, it was not possible to distinguish between the effect of –*413AA* or (*GT*)_31,32_(*GT*)_31,32_ homozygosity because of the very low frequency of subjects in whom these two alleles were not found together (Table 2).

Distribution of –413A>T *HMOX1* SNP alleles in endometriosis and control group subjects was found to be similar to distribution reported for other populations [28,29] and is consistent with data presented in HapMap database (http://hapmap.ncbi.nlm.nih.gov/, accessed on 21 March 2021). Analysis of the frequency of *HMOX1* (*GT*)_n_ alleles has revealed two clearly different groups displaying 23–24 repeats or 31–32 (*GT*) repeats which account for ca. 80% of all studied subjects. This observation is also consistent with other reports on (*GT*)_n_ allele distribution [30]. It should be stressed, that in a majority of these studies (*GT*)_n_ alleles were classified into short or long and sometimes medium variants. However, criteria for such classification were arbitrary, and therefore, to avoid a bias related to a great diversity of (*GT*)_n_ variants, we decided to limit our analysis only to specific long (*GT*)_31,32_ and short (*GT*)_23,24_ alleles.

Our present result in women with endometriosis is similar to the previous reports of Ono et al. [28,29] who found that prevalence of –*413A HMOX1* homozygote is associated with a higher risk of women’s’ hypertension and coronary artery disease. In respect to the (*GT*)_n_ *HMOX1* polymorphism, our results are also similar to results reported for a variety of other diseases including some cardiovascular disorders and transplanted organ dysfunction [30] as well as idiopathic recurrent miscarriage [39], where longer (*GT*)_n_ variants (n>30) were associated with increased susceptibility to the disease.

The biological significance of *HMOX1* promoter polymorphism still remains a matter of controversy. It is generally accepted that short (*GT*)_n_ variants are associated with an increased HMOX1 expression and activity. This is based on both the biological observations of cell lines isolated from subjects carrying different (*GT*)_n_ variants [40,41] and cloned *HMOX1* promoter/luciferase assays [42,43]. It should be stressed, however, that these studies were focused on (*GT*)_n_ variants and did not take into consideration a possible influence of −413A>T SNP polymorphism and possibly other yet-unknown factors. In contrast to these reports, luciferase reporter assays performed by Ono et al. [29] have revealed that transcriptional activity of −413*A*(*GT*)_30_-harboring *HMOX1* promoter constructs was about six times higher than that of −413*T*(*GT*)_23_ ones. This strongly implies that the relationship between the length of (*GT*)_n_ variants and prevalence of −413A>T alleles needs further elucidation.

Expression of *HMOX1* may be stimulated by inflammatory cytokines as well as oxidative stress agents [22,23,24] which are known to be involved in the pathogenesis of endometriosis [12,13,14,15]. Oxidative stress agents may include heme [17] which may be released into the peritoneal cavity and the lumen of endometriotic ovarian cysts (chocolate cysts) from erythrocytes originating from retrograde menstruation and shedding of endometriotic tissue in course of menstrual cycle. It should be stressed, however, that final elucidation of the regulatory mechanism(s) of HMOX1 expression in endometriosis requires more extensive studies.

The specific role of HMOX1 in etiopathogenesis of endometriosis also remains to be established. There is a growing evidence that this enzyme may exert a variety of cytoprotective effects [22,23,24]. HMOX1 was reported to protect cells from apoptosis [44,45,46] and may exert anti-inflammatory effects [47,48,49,50]. This effect may be mediated by degradation of toxic and pro-oxidant heme, as well as in generation of products of heme metabolism such as biliverdin and bilirubin which were also reported to exert anti-inflammatory and immunoregulatory activity [51,52,53]. Therefore, it is possible that an increased HMOX1 activity in endometriotic cells might protect them from heme toxicity and oxidative stress and thus facilitate their survival and adhesion in ectopic sites. Furthermore, HMOX1 plays an important role in angiogenesis [24,54,55], thus further supporting its possible role in development of endometriotic lesions.

Clinical significance of *HMOX1* promoter polymorphism in endometriosis also remains obscure. It would be of interest to correlate *HMOX1* polymorphism with some clinical features of endometriosis such as stage of the disease, lesion type and their location. However, the patients’ cohort recruited in the present study consisted mostly (95%) of women with moderate/severe (III/IV) stage of the disease. Furthermore, 95% of patients had endometriotic ovarian cysts, and only 5% of them had peritoneal lesions. Such patients’ stratification made any statistical correlation analysis meaningless. Therefore, further studies on a group of patients including significantly more cases with minimal/mild stage of endometriosis and higher prevalence of only peritoneal lesions would be necessary.

In summary, our present results show that endometriosis is associated with functional polymorphism of *HMOX1* gene promoter. This suggests that HMOX1 plays an important role in etiopathogenesis of this disorder, possibly by exerting a protective effect and facilitating survival of endometriotic cells in ectopic sites. This, however, needs further extensive studies.

## Figures and Tables

**Table 1:** Allele and genotype distribution of −413A>T SNP (single-nucleotide polymorphism) and two most frequent (*GT*)_n_ dinucleotide repeat variants of *HMOX1* gene in patients with endometriosis and control women.

	Allele/Genotype	Endometriosis (*n* = 228)	Control (*n* = 415)	*p*-Value *	OR (95% CI)
-413A>T	A T	269 (59.0%) 187 (41.0%)	459 (55.3%) 371 (44.7%)	0.2017	1.16 (0.92–1.47)
	AA AT TT	90 (39.5%) 89 (39.0%) 49 (21.5%)	127 (30.6%) 205 (49.4%) 83 (20.0%)	0.0228 0.0132 0.6547	1.48 (1.06–2.07) 0.66 (0.48–0.92) 1.09 (0.74–1.63)
	HWE *P*	0.004	0.987		
(*GT*)_n_	(*GT*)_23,24_ (*GT*)_31,32_	141 (30.8%) 240 (52.4%)	262 (31.6%) 392 (47.2%)	0.777 0.0754	0.96 (0.75–1.23) 1.23 (0.98–1.55)
	(*GT*)_23,24_(*GT*)_23,24_ (*GT*)_23,24_(*GT*)_31,32_ (*GT*)_31,32_(*GT*)_31,32_	23 (10.1%) 62 (27.2%) 72 (31.6%)	41 (9.1%) 119 (28.7%) 101 (24.3%)	0.9203 0.6891 0.0480	1.02 (0.60–1.75) 0.93 (0.65–1.33) 1.43 (1.01–2.05)
	HWE *P*	0.09 **	0.39 **		

* p-values were calculated by Pearson χ^2^ test. ** HWE analysis was performed with all (*GT*)_n_ genotypes found in endometriosis and control group (for details see Table 2). OR (95% CI): odds ratio (95% confidence intervals). *n*: number of cases. HWE. *p*: Probability for Hardy–Weinberg equilibrium.

**Table 2:** Distribution of (*GT*)_n_ variants in relation to −416A>T genotypes of *HMOX1* gene in women with endometriosis and control subjects.

(*GT*)_n_ Genotype		416A>T SNP Genotype					
		AA		AT		TT	
		Endometriosis *n* = 90	Control *n* = 127	Endometriosis *n* = 89	Control *n* = 205	Endometriosis *n* = 49	Control *n* = 83
21	23	0	0	1 (0.4%)	0	0	1 (0.2%)
21	24	0	0	0	1 (0.2%)	1 (0.4%)	0
21	26	0	0	0	0	1 (0.4%)	0
21	31	0	0	0	1 (0.2%)	0	0
21	32	0	0	0	1 (0.2%)	0	0
22	27	0	0	0	0	1 (0.4%)	0
22	31	0	0	1 (0.4%)	1 (0.2%)	0	0
23	23	0	0	0	1 (0.2%)	11 (4.8%)	16 (3.9%)
23	24	0	0	1 (0.4%)	0	6 (2.6%)	21 (5.1%)
23	25	0	0	3 (1.3%)	2 (0.5%)	2 (0.9%)	6 (1.4%)
23	26	0	0	0	1 (0.2%)	1 (0.4%)	2 (0.5%)
23	27	0	0	0	1 (0.2%)	0	1 (0.2%)
23	28	0	0	0	2 (0.5%)	0	1 (0.2%)
23	29	0	0	0	0	2 (0 (1.9%)	1 (0.2%)
23	30	0	0	1 (0.4%)	10 (2.4%)	0	0
23	31	2 (0.9%)	1 (0.2%)	34 (14.9%)	74 (17.8%)	1 (0.4%)	0
23	32	0	0	8 (3.5%)	9 (2.1%)	0	0
23	33	0	0	0	1 (0.2%)	0	0
23	35	0	0	0	0	1 (0.4%)	2 (0.5%)
23	37	0	0	0	0	2 (0.9%)	3 (0.7%)
23	38	0	0	0	0	4 (1.7%)	8 (1.9%)
23	39	0	0	0	0	1 (0.4%)	0
24	24	0	0	0	0	5 (2.2%)	3 (0.7%)
24	25	0	0	0	1 (0.2%)	5 (2.2%)	1 (0.2%)
24	26	0	0	0	1 (0.2%)	0	2 (0.5%)
24	28	0	0	1 (0.4%)	1 (0.2%)	0	0
24	29	0	0	1 (0.4%)	1 (0.2%)	0	0
24	30	0	0	1 (0.4%)	4 (1%)	1 (0.4%)	0
24	31	0	0	15 (6.6%)	30 (7.2%)	0	0
24	32	0	0	2 (0.9%)	5 (1.2%)	0	0
24	38	0	0	0	0	3 (1.3%)	6 (1.4%)
24	39	0	0	0	0	1 (0.4%)	0
24	40	0	0	0	0	0	1 (0.2%)
25	27	0	0	0	0	0	1 (0.2%)
25	28	0	0	0	0	0	1 (0.2%)
25	30	0	0	1 (0.4%)	2 (0.5%)	0	0
25	31	3 (1.3%)	2 (0.5%)	3 (1.3%)	5 (1.2%)	0	0
25	32	2 (0.9%)	0	1 (0.4%)	1 (0.2%)	0	0
25	34	0	0	0	1 (0.2%)	0	0
25	37	0	0	0	1 (0.2%)	0	2 (0.5%)
25	39	0	0	1 (0.4%)	0	0	1 (0.2%)
26	29	1 (0.4%)	0	0	0	0	0
26	31	1 (0.4%)	1 (0.2%)	0	3 (0.7%)	0	0
26	32	0	0	0	1 (0.2%)	0	0
27	31	1 (0.4%)	2 (0.5%)	1 (0.4%)	4 (1%)	0	0
27	32	0	1 (0.2%)	0	0	0	0
28	31	2 (0.9%)	4 (1%)	2	1 (0.2%)	0	0
28	32	0	1 (0.2%)	0	0	0	0
29	29	1 (0.4%)	0	0	0	0	0
29	30	0	1 (0.2%)	0	0	0	0
29	31	2 (0.9%)	5 (1.2%)	1 (0.4%)	0	0	0
29	38	0	0	0	0	0	1 (0.2%)
30	30	0	5 (1.2%)	0	0	0	0
30	31	1 (0.4%)	3 (0.7%)	1 (0.4%)	1 (0.2%)	0	0
30	32	1 (0.4%)	2 (0.5%)	0	0	0	0
30	35	0	0	0	1 (0.2%)	0	0
30	38	0	0	0	4 (1%)	0	0
31	31	50 (21.9%)	72 (17.3%)	0	3 (0.7%)	0	0
31	32	22 (9.6%)	19 (4.6%)	0	1 (0.2%)	0	0
31	33	0	2 (0.5%)	0	0	0	0
31	34	1 (0.4%)	0	0	0	0	0
31	35	0	0	1 (0.4%)	0	0	0
31	36	0	0	0	1 (0.2%)	0	0
31	37	0	0	2 (0.9%)	9 (2.2%)	0	0
31	38	0	0	4	15 (3.6%)	0	0
31	40	0	0	0	1 (0.2%)	0	0
32	32	0	6 (1.4%)	0	0	0	0
32	37	0	0	1 (0.4%)	1 (0.2%)	0	0
32	38	0	0	1 (0.4%)	0	0	0
32	40	0	0	0	1 (0.2%)	0	0
36	38	0	0	0	0	0	1 (0.2%)
38	38	0	0	0	0	0	1 (0.2%)

Highlighted are the most frequent genotypes. *n*: number of cases.

**Table 3:** Distribution of two major *HMOX1* −413A>T (*GT*)_n_ haplotypes and genotypes in patients with endometriosis and control women.

Haplotype/Genotype	Endometriosis (*n* = 228)	Control (*n* = 415)	*p*-Value *	OR (95%CI)
−*413A*(*GT*)_31,32_ −*413T*(*GT*)_23,24_	238 (52.1%) 138 (30.2%)	388 (46.8%) 260 (31.3%)	0.0381 0.6954	1.27 (1.01–1.60) 0.95 (0.74–1.15)
−*413A*(*GT*)_31,32_/*413A*(*GT*)_31,32_ −*413A*(*GT*)_other_/−*413A*(*GT*)_other_	72 (31.6%) 18 (7.9%)	97 (23.4%) 30 (7.2%)	0.0238 0.7641	1.51 (1.06–2.17) 1.10 (0.60–2.02)

* *p*-Values were calculated by Pearson χ^2^ test or Fisher exact probability test where applicable. OR (95% CI): odds ratio (95% confidence intervals). *n*: number of cases.

## Data Availability

All data are included in the paper.
